# Impact of Influenza Vaccination on All-Cause Mortality and Hospitalization for Pneumonia in Adults and the Elderly with Diabetes: A Meta-Analysis of Observational Studies

**DOI:** 10.3390/vaccines8020263

**Published:** 2020-05-30

**Authors:** Angela Bechini, Alessandra Ninci, Marco Del Riccio, Ilaria Biondi, Jacopo Bianchi, Paolo Bonanni, Edoardo Mannucci, Matteo Monami

**Affiliations:** 1 Department of Health Sciences, University of Florence, 50134 Florence, Italy; angela.bechini@unifi.it (A.B.); alessandra.ninci@unifi.it (A.N.); ilaria.biondi@unifi.it (I.B.); jacopo.bianchi@unifi.it (J.B.); paolo.bonanni@unifi.it (P.B.); 2 Diabetology, Careggi Hospital and University of Florence, 50134 Florence, Italy; edoardo.mannucci@unifi.it (E.M.); matteo.monami@unifi.it (M.M.)

**Keywords:** influenza vaccination, diabetes, hospitalization, mortality, meta-analysis

## Abstract

Diabetes is a chronic condition that can be worsened by complications such as seasonal influenza virus infections. The aim of the present meta-analysis is the systematic retrieval and analysis of all available evidence on the effects of an influenza vaccine on diabetic patients. We conducted a systematic review and meta-analysis by searching MEDLINE, Embase and the Cochrane databases from inception until April 2019. We included all types of studies reporting on the effectiveness of influenza vaccination in adult and elderly patients with type 1 and type 2 diabetes. The Newcastle-Ottawa scale was used to assess risk of bias, the GRADE methodology was used to assess the evidence for each outcome. A total of 2261 studies were identified, of those, 6 studies completely fulfilled the inclusion criteria. In the 6 studies included in the analysis, influenza vaccination was associated with a lower mortality rate (Mantel Haenszel Odds Ratio (MH-OR), 95% CI: 0.54 (0.40; 0.74), *p* < 0.001). Patients who received influenza vaccination showed a lower risk of hospitalization for pneumonia (MH-OR, 95% CI: 0.89; (0.80; 0.98), *p* = 0.18). A sensitivity analysis using fixed effect model confirmed the results (MH-OR, 95% CI: 0.91; (0.87; 0.96); *p* = 0.001). The results of this meta-analysis are clinically relevant and support the recommendation for all persons with diabetes to receive influenza vaccination.

## 1. Introduction

Seasonal influenza virus infections are often associated with mild and self-limiting symptoms without any need for medical care. However, in some subjects, and particularly in those with advanced age and/or comorbidities, influenza can be aggravated by complications [1]. Several studies have shown that influenza in patients with diabetes is associated with a higher mortality and risk of complications, such as deep venous thrombosis, pulmonary embolism, and pneumonia [2]. Because of this higher risk [3], influenza vaccination is highly recommended in patients with diabetes by the World Health Organization (WHO) [4] and by several guidelines from scientific societies [5,6,7].

One of the possible mechanisms underlying the greater susceptibility of diabetic patients to influenza and its complications could be the impaired immune response which is related to glucotoxicity [8,9,10]. Glucose toxicity could theoretically reduce the immune response to vaccines: in fact, some studies have shown a reduction in the innate immune response to vaccination [11,12] in patients with diabetes. Nevertheless, some others reported that the humoral response was not dissimilar from the response of healthy subjects [13,14]. The efficacy of vaccination in preventing influenza infection and its complications in patients with diabetes needs, therefore, to be specifically assessed.

Several observational studies reported that mortality and hospitalization rates are significantly different in vaccinated diabetic patients, when compared to non-vaccinated patients. The aim of the present meta-analysis is the systematic retrieval and analysis of all available evidence on the effects of influenza vaccine on the risk of hospitalization for pneumonia and mortality in patients with diabetes.

## 2. Materials and Methods

### 2.1. Protocol and Registration

Methods of the present meta-analysis and inclusion criteria were specified in advance in a protocol, available on the PROSPERO website (CRD42019127620; https://www.crd.york.ac.uk/PROSPERO/), according to the criteria of the PRISMA statement [15].

### 2.2. Eligibility Criteria

To be eligible for this systematic review, a study had to be an original report assessing the effectiveness of influenza vaccination in adult diabetic patients (type 1 and type 2). No studies based on pediatric patients were considered eligible for inclusion. The main endpoints were all-cause mortality and hospitalization for pneumonia. The non-exposed or control group were unvaccinated diabetic patients. Only observational (cohort or nested case-control) studies were included.

### 2.3. Study Search and Selection

Studies were identified by searching MEDLINE, EMBASE, and Cochrane databases for studies in English, up to April 24th, 2019, on diabetic subjects exposed to influenza vaccination. Detailed information on the search string is reported in the Supplementary Materials (Table S1).

The eligibility assessment was performed in two stages. In the first one, articles were screened by title and abstract in a blinded manner by two couples of authors (I.B. and M.D.R or A.N. and J.B.). Disagreements were resolved by consensus and, if not reachable, by a third investigator (M.M. or A.B.). In the second stage, the entire text of the selected articles were read by four authors (I.B., M.D.R., A.N. and J.B.).

### 2.4. Data Retrieval

We developed a data extraction spreadsheet to collect data. A.N. and M.D.R extracted the data from eligible studies and J.B. and I.B. checked the extracted data. Disagreements were solved by M.M.

The following parameters/information were extracted: (1) Study identification characteristics; title, first author, year of publication; (2) characteristics of participants; (3) type of intervention; (4) type of outcome measure. Risk ratios and odds ratios were extracted directly or calculated from the publications. The quality of trials was assessed using the parameters proposed by the Newcastle-Ottawa Scale (NOS) [16].

### 2.5. Statistical Analyses

Meta-analysis was performed using the Mantel-Haenszel odds ratio (MH-OR) with 95% confidence interval (95% CI); it was calculated for all outcomes defined above, on an intention-to-treat basis. Heterogeneity was assessed by using I2 statistics. A random effects model was applied in the primary analysis, whereas fixed effect models were applied for sensitivity analysis. Begg and Mazumdar’s test was performed in order to estimate possible publication/disclosure bias. All analyses specified above were performed using Review Manager 5.3; Copenhagen: The Nordic Cochrane Centre, The Cochrane Collaboration, 2014. The GRADE methodology [17] was used to assess the quality of the body of retrieved evidence, using the GRADEpro GDT software (GRADEpro Guideline Development Tool, McMaster University, 2015. Available from gradepro.org).

### 2.6. Role of the Funding Source

This research was performed as a part of the institutional activity of the unit, with no specific funding. All expenses, including salaries of the investigators, were covered by public research funds.

## 3. Results

### 3.1. Studies Selection

**Figure 1:**
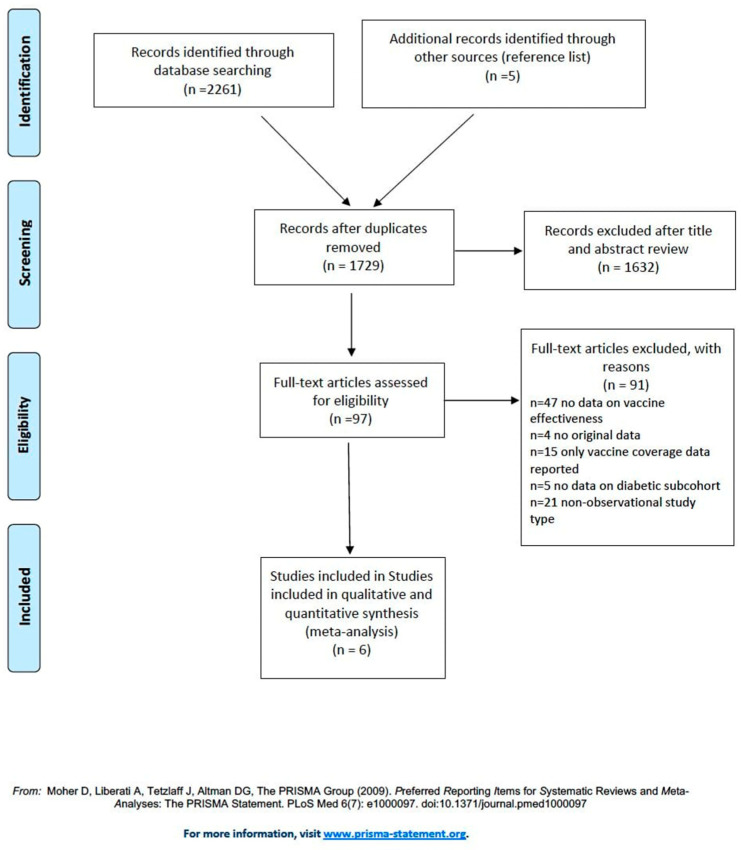
Flow diagram for the systematic review (PRISMA statement [15]).

A total of 2261 studies (720 from Medline, 1332 from Embase and 209 from Cochrane) were identified; five additional records were identified by checking a reference list (cross-reference search) [18]. After removing 537 duplicates, 1632 studies were excluded after title and abstract review. The full text of the remaining items was examined in detail. Six studies fulfilled the inclusion criteria (Figure 1). None of the studies include pregnant women.

The main characteristics of those studies are summarized in Table 1. The main reasons for exclusion were: No data on vaccine effectiveness in diabetic patients reported in the study (*n* = 47), no new original data (*n* = 4), studies that only reported vaccine coverage data (*n* = 15), absence of data on diabetic subcohort (*n* = 5), and non-observational study type (*n* = 21).

The quality of the included studies was high, except for studies [21] and [19] (Table 2 and Table 3).

### 3.2. All Cause-Mortality

In the 6 studies included in the analysis, influenza vaccination was associated with a lower mortality rate (MH-OR, 95% CI: 0.54 (0.40; 0.74), *p* < 0.001) and this result was confirmed in a sensitivity analysis using a fixed effect model (MH-OR, 95% CI: 0.67 (0.64; 0.70); *p* < 0.001) (Table 4). No publication bias was detected using Begg and Mazumdar’s test (Kendall’s tau without continuity correction: 0.05, *p* = 0.88). I2 statistics suggested a relevant heterogeneity (I2: 96, *p* < 0.001).

### 3.3. Hospitalization for Pneumonia

Four studies reported information on hospitalization for pneumonia. Patients who received influenza vaccination showed a lower risk of hospitalization for pneumonia (MH-OR, 95% CI: 0.89; (0.80; 0.98), *p* = 0.18). A sensitivity analysis using fixed effect model confirmed the results (MH-OR, 95% CI: 0.91, (0.87; 0.96); *p* = 0.001) (Table 5). No publication bias was detected using Begg and Mazumdar’s test (Kendall’s tau without continuity correction: −0.33, *p* = 0.50). I2 statistics suggested a relevant heterogeneity (I2: 62.9%, *p* = 0.044).

### 3.4. Quality of Evidence

Using the GRADE algorithm, the overall quality of evidence was rated “moderate” for both endpoints (Table 6).

## 4. Discussion

The results of the present meta-analysis are consistent with those of a previous systematic review [25], which considered some articles included in the present work (Heymann [19], Rodriguez-Blanco [20], Schade [21], Looijmans-Vanden Akker [23], Wang [24]). We excluded some studies included in the previous meta-analysis because they did not report separate data on hospitalization for pneumonia and mortality (Gasparini [26], Selvais [27], Hak [28], Colqhoun [29], Lau [30], Isotani [31]). In comparison with that meta-analysis, the present work also includes one large retrospective cohort study [22], which was not yet available at the time of the previous review [25].

The strengths of this study—as reported by Vamos et al.—include the use of a large population-based cohort of patients with type 2 diabetes, long follow-up time, the availability of key laboratory and clinical parameters, and knowing the exact dates of vaccination [22].

The wide reduction of all-cause mortality associated with influenza vaccination in diabetic patients, shown in the results of this meta-analysis, is consistent with the reduction reported in elderly people [32]. Such a relevant effect can be partly explained by the reduction of complications of influenza, such as pneumonia. As a matter of fact, in the present meta-analysis influenza vaccination was associated with a reduced hospitalization rate for pneumonia. However, other mechanisms could also be involved. Epidemiological evidence suggests that in patients with cardiovascular disease, influenza vaccination may reduce cardiovascular mortality and combined cardiovascular events [33]. Infections such as influenza can produce a transient but relevant increase in blood glucose levels; this could worsen diabetes and its intercurrent complications [34].

It is important to mention that the included studies assess the effectiveness of influenza vaccination in countries where vaccination coverages are diverse. In European countries vaccination is generally recommended in the elderly, adults with chronic conditions or healthcare workers to reduce transmission [35], but influenza vaccination coverage rates are generally still not adequate [36]. In England, where influenza vaccine is offered to all people with chronic conditions, Vamos et al. found that vaccination coverage ranged from 63.1% in 2008/09 to 69.0% in 2006/07 [22]. In the study by Rodriguez-Blanco et al., annual influenza vaccine coverages ranged from 59.8% to 71.3% [20], and these findings are consistent with other data reported in older people with diabetes in Spain and other developed countries, where approximately 60–70% of these subjects are immunized annually against influenza [37,38]. The Netherlands represent an exception, since it reached the highest reported vaccination coverage rates among older age groups (82% in 2008/09) compared to other EU countries [39]. In the USA, estimated influenza vaccination levels for persons aged >65 years in 1997, 1999, and 2001 were 63.2%, 65.7%, and 63.0%, respectively, and the estimated influenza coverage among diabetic patients in the study population was 36% in 1996 and 37.2% in 1997 [40]. In Israel, influenza vaccination rates increased in the elderly (aged 65 years and over) in the 2000/2001 (54.4%) and 2001/2002 (60.8%) winter seasons, compared to the previous two seasons (51.6% in 1998/1999 and 49.1% in 1999/2000) [41]. In Taiwan, where influenza vaccination is offered free of charge to high-risk and elderly individuals since 1998, the influenza vaccination rates for elderly subjects were still lower than 50% in 2007 [42].

Several limitations of the present meta-analysis should be recognized. Collected data are derived from observational retrospective studies, which, although well designed and properly executed, cannot completely avoid the risk of confounding. Patients with diabetes who choose to get vaccinated against influenza could differ in some respects from those who, despite current recommendations, decide to avoid the vaccine. Some of these confounders (e.g., health beliefs, self-care, adherence to physicians’ prescriptions, etc.) cannot be properly measured and adjusted for in retrospective studies. This could lead to an overestimation of the effectiveness of the vaccination.

The retrospective nature of the studies included in the meta-analysis limits the reliability of the outcome diagnosis. Although “all-cause mortality” poses no such problem, “hospitalization for pneumonia” is based on a diagnosis performed without following any pre-determined criteria. For the same reason, influenza-related mortality was not considered as an outcome, because of the elevated risk of misclassifications and misdiagnosis.

Another relevant limitation is represented by the heterogeneity of the results, which is confirmed by I2 statistics. Unfortunately, the number of available studies is not sufficient to explore (either through analyses of subgroups of studies, or through meta-regressions) the factors underlying this heterogeneity. Pneumococcal vaccination besides influenza vaccination are recommended to diabetic patients, therefore, we might expect that variations in pneumococcal vaccination status could influence our outcomes [43]. Further hypothetical reasons for discrepancies across studies can include differences in population characteristics, differences in case mix, heterogeneity of vaccine formulations and diversities in the biological behaviour of strains of influenza virus in different years.

## 5. Conclusions

Despite all these limitations, the results of this meta-analysis are clinically relevant and support the recommendation of influenza vaccination in diabetic patients as a key mean to reduce all-cause mortality and hospitalization due to the infection [7,44,45,46].

The present meta-analysis shows that influenza vaccination is associated with a relevant reduction of complications of the infection, despite some evidence indicating that the immune response to vaccination is reduced in persons with diabetes [11,12]. Nonetheless, other studies have shown that the humoral immune response after influenza vaccination in diabetic patients is not dissimilar from that of the general population [13,14]. Increased HbA1c may be associated with decreased NKG2D receptor, which could be the cause of increased susceptibility of influenza infection in diabetic patients [47]. Influenza vaccination is therefore needed to reduce influenza infection in diabetics.

Nowadays, many types of vaccines against influenza are available on the market. While any influenza vaccine can be crucial to avoid complications in risk groups, the use of the most appropriate vaccine could optimize the result in preventing hospitalizations and death [36]. Influenza vaccination coverage has doubled in 56 countries (influenza vaccine distribution is expressed as the number of doses distributed/1000 total population each year) [48]. This worldwide increase in influenza vaccination uptake reflects greater understanding of the burden of influenza disease and the effectiveness of influenza vaccination, particularly in high-risk patients.

The global use of influenza vaccine will almost certainly continue to increase, even in non- vaccine-producing countries, and this changing pattern in the distribution of influenza vaccine will have important implications for the next pandemic.

## Figures and Tables

**Table 1:** Characteristics of included studies on influenza vaccine effectiveness in diabetic patients.

Author	Study Design and Period	Country	Age in Years (Mean/Range)	Male (%)	Identification of Diabetic Patient and Data Source	Circulating Influenza (Sub)Strains	Study Size (*n*)
**Cohort studies**							
Heymann, 2004 [19]	Retrospective, 2000/2001	Israel	Vacc ^1^., 72.8 Non vacc., 73.1	Vacc. 51.8 Non vacc 42.1	International Classification of Diseases (ICD)-9 codes, diabetes register of healthcare service	Not reported	16,383
Rodriguez-Blanco, 2012 [20]	Retrospective, 2002–2005	Spain	Vacc., 75.2 Non Vacc 73.1	Vacc., 39.8 Non vacc., 42.2	ICD-9 codes, clinical records	A(H3N2)	2650
Shade, 2000 [21]	Retrospective, 1996–1998	US	65 to 114 years	Not reported	ICD-9 codes, hospital discharge	Not reported	26,443 in 96/97 23,839 in 97/98
Vamos, 2016 [22]	Retrospective 2003–2010	England	Vacc., 66.0 Non vacc., 56.2	Vacc., 53.9 Non vacc., 54.2	ICD-10 codes, hospital admissions	A(H1N1) pdm09	124,503
**Case-control studies**							
Looijmans, 2006 [23]	Nested, 1999/2000	Netherlands	Cases, 68.1 Controls 69.8	Cases 51.6 Controls 38.3	International Classification of Primary Care (ICPC) codes, GPs	A(H3N2)	1753 (192 cases, 1561 controls)
Wang, 2013 [24]	Nested, 2001–2009	Taiwan	Vacc., 73.1 Non vacc. 73.2	Vacc., 50.0 Non vacc., 49.5	ICD-9 codes, NHRI-database	Not reported	9025, (4571 vacc., 4454 non vacc.)

^1^ Vacc: vaccinated.

**Table 2:** Quality of the cohort studies included in the meta-analysis (NOS Scale).

Cohort Study										
Author, Year	Selection				Comparability		Outcome			Total Quality Score
	Representativeness of the Exposed Cohort	Selection of the Non-Exposed Cohort	Ascertainment of Exposure	Demonstration that Outcome of Interest Was not Present at Start of Study	Main Factor	Additional Factor	Assessment of Outcome with Independency	Adequacy of Follow up Length (to Asses Outcome)	Loss to Follow up Acceptable (Less than 10% and Reported)	
Heymann, 2004 [19]	*	*	*	*	*		*			6/9
Rodriguez-Blanco, 2012 [20]	*	*	*	*	*	*	*	*	*	9/9
Shade, 2000 [21]	*	*			*		*			4/9
Vamos, 2016 [22]	*	*	*	*	*	*	*	*	*	9/9

**Table 3:** Quality of the case-control studies included in the meta-analysis (NOS Scale).

Case-Control Study										
Author, Year	Selection				Comparability		Exposure			Total Quality Score
	The Case Definition Adequate	Representative Series of Cases	Selection of Controls	Definition of Controls	Main Factor	Additional Factor	Ascertainment of Exposure Using Secure Records	Ascertainment of Exposure Using the Same Method for Cases and Controls	Ascertainment of Exposure with Non-Response Rate for Both Groups	
Looijmans, 2006 [23]	*	*	*	*	*	*	*	*	*	**9/9**
Wang, 2013 [24]	*	*	*	*	*	*	*	*	*	**9/9**

**Table 4:** Forest plot of the meta-analysis of all cause-mortality rate.

**Study Name**	**Statistics for Each Study**					**Rate Ratio and 95% IC**
	**Rate Ratio**	**Lower Limit**	**Upper Limit**	***Z*-Value**	***p*-Value**	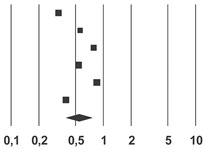 Favors Vaccination Favors Non-Vaccination
Heymann, 2004 [19]	0.33	0.25	0.43	−8.41	0.00	
Looijmans, 2006 [23]	0.56	0.32	0.97	−2.06	0.04	
Rodriguez-Blanco, 2012 [20]	0.78	0.53	1.15	−1.24	0.21	
Shade, 2000 [21]	0.54	0.50	0.58	−16.08	0.00	
Vamos, 2016 [22]	0.85	0.80	0.90	−5.13	0.00	
Wang, 2013 [24]	0.39	0.32	0.48	−9.10	0.00	
**Overall**	0.54	0.40	0.74	−3.84	0.00	

**Table 5:** Forest Plot of metanalysis of hospitalization for pneumonia rate.

**Study Name**	**Statistics for Each Study**					**Rate Ratio and 95% IC**
	**Rate Ratio**	**Lower Limit**	**Upper Limit**	***Z*-Value**	***p*-Value**	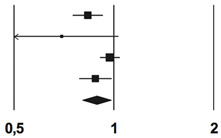 Favors Vaccination Favors Non-Vaccination
Heymann, 2004 [19]	0.83	0.75	0.92	−3.34	0.01	
Looijmans, 2006 [23]	0.70	0.47	1.03	−0.80	0.072	
Vamos, 2016 [22]	0.97	0.91	1.04	−0.77	0.00	
Wang, 2013 [24]	0.88	0.78	0.99	−2.20	0.00	
**Overall**	0.89	0.80	0.98	−2.36	0.00	

**Table 6:** GRADE evidence for influenza vaccination on all-cause mortality and hospitalization for pneumonia. Influenza vaccine compared to no vaccine for diabetes.

Certainty Assessment							N° of Patients		Effect		Certainty	Importance
*N* of Studies	Study Design	Risk of Bias	Inconsistency	Indirectness	Imprecision	Other Considerations	Influenza Vaccine	No Vaccine	Relative (95% CI)	Absolute (95% CI)		
**All-cause mortality**												
6	Observational studies	Serious ^a^	Very serious ^b^	Not serious	Not serious	Strong association all plausible residual confounding would reduce the demonstrated effect	3545/108241 (3.3%)	5195/88837 (5.8%)	OR 0.54 (0.40 to 0.74)	26 fewer per 1.000 (from 34 fewer to 15 fewer)	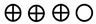 MODERATE	CRITICAL
**Hospitalization for pneumonia**												
4	Observational studies	Serious ^a^	Very serious ^b^	Not serious	Not serious	Strong association all plausible residual confounding would reduce the demonstrated effect	3203/83990 (3.8%)	2702/57038 (4.7%)	OR 0.89 (0.80 to 0.98)	5 fewer per 1.000 (from 9 fewer to 1 fewer)	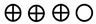 MODERATE	IMPORTANT

^a^ Possible confounding bias; ^b^ elevated heterogeneity across studies, as demonstrated by 12 statistics. Differences in case mix, populations and types of vaccines.

## Supplementary Materials

The following are available online at https://www.mdpi.com/2076-393X/8/2/263/s1, Table S1: Search strategies.
