# Supplementary material for: Impact of Influenza Vaccination on All-Cause Mortality and Hospitalization for Pneumonia in Adults and the Elderly with Diabetes: A Meta-Analysis of Observational Studies
Source: Vaccines (Basel). 2020 May 30;8(2):263. doi: 10.3390/vaccines8020263 (PMC7349976; doi:10.3390/vaccines8020263)
Supplement: Supplementary file 1 [file vaccines-08-00263-s001.pdf]

Table S1. Search strategies.

**PubMed**<br>("Metabolic Diseases"[Mesh] OR (metabolic disease[tw] OR metabolic diseases[tw]) OR (metabolic disorder[tw] OR metabolic disordered[tw] OR metabolic disorders[tw]) OR metabolic syndrome[tw] OR syndrome X[tw] OR MetS[tw] OR "Diabetes Mellitus"[Mesh] OR (diabet[tw] OR diabeta[tw] OR diabeta'[tw] OR diabetacorum[tw] OR diabetaction[tw] OR diabetaeta[tw] OR diabetagenic[tw] OR diabetamid[tw] OR diabetas[tw] OR diabetc[tw] OR diabetcieza[tw] OR diabetcs[tw] OR diabete[tw] OR diabete's[tw] OR diabetea[tw] OR diabetec[tw] OR diabeted[tw] OR diabetees[tw] OR diabeteic[tw] OR diabeteics[tw] OR diabetelogical[tw] OR diabetelogy[tw] OR diabetemellitus[tw] OR diabeter[tw] OR diabeterelated[tw] OR diabetergestemd[tw] OR diabeters[tw] OR diabetes[tw] OR diabetes'[tw] OR diabetes''[tw] OR diabetes'african[tw] OR diabetes'data[tw] OR diabetes'risk[tw] OR diabetes's[tw] OR diabetes1[tw] OR diabetes11[tw] OR diabetes2[tw] OR diabetes3[tw] OR diabetes39[tw] OR diabetesa[tw] OR diabetesand[tw] OR diabetesassociated[tw] OR diabetesatlas[tw] OR diabetesbaicalin[tw] OR diabetesbased[tw] OR diabetesbb[tw] OR diabetesbut[tw] OR diabetescancerconnect[tw] OR diabetescardiac[tw] OR diabetescare[tw] OR diabetescarvone[tw] OR diabetescoach[tw] OR diabetescould[tw] OR diabetescpme[tw] OR diabetesde[tw] OR diabetesdriving[tw] OR diabetesdue[tw] OR diabetese[tw] OR diabeteseducation[tw] OR diabeteses[tw] OR diabetesexercise[tw] OR diabetesexercised[tw] OR diabetesforeningen[tw] OR diabetesforms[tw] OR diabetesgenes[tw] OR diabetesgesellschaft[tw] OR diabeteshad[tw] OR diabetesii[tw] OR diabetesimpact[tw] OR diabetesin[tw] OR diabetesincidencia[tw] OR diabetesindia[tw] OR diabetesinduced[tw] OR diabetesinsipidus[tw] OR diabetesinsulin[tw] OR diabetesis[tw] OR diabeteskockazat[tw] OR diabetesl[tw] OR diabetesliiton[tw] OR diabeteslike[tw] OR diabeteslive[tw] OR diabetesmeds[tw] OR diabetesmellitus[tw] OR diabetesmine[tw] OR diabetesmortalitas[tw] OR diabetesoutcomequality[tw] OR diabetespatienten[tw] OR diabetespatients[tw] OR diabetesplanning[tw] OR diabetespravention[tw] OR diabetespresenting[tw] OR diabetesprevalencia[tw] OR diabetespro[tw] OR diabetesproduced[tw] OR diabetesprone[tw] OR diabetesrask[tw] OR diabetesrelated[tw] OR diabetesrisks[tw] OR diabetesrobert[tw] OR diabetess[tw] OR diabetesscenariosforjuniordoctors[tw] OR diabetesschulung[tw] OR diabetessoftware[tw] OR diabetesspecific[tw] OR diabetesstation[tw] OR diabetesstudy[tw] OR diabetessystematic[tw] OR diabetesthe[tw] OR diabetestherapie[tw] OR diabetesthere[tw] OR diabetestrade[tw] OR diabetestrial[tw] OR diabetesvereniging[tw] OR diabetesweekly[tw] OR diabetesxglucose[tw] OR diabeteszentrum[tw] OR diabetetes[tw] OR diabetetic[tw] OR diabetetogenesis[tw] OR diabetetogenic[tw] OR diabetets[tw] OR diabetex[tw] OR diabetgic[tw] OR diabethic[tw] OR diabeti[tw] OR diabetia[tw] OR diabetic[tw] OR diabetic'[tw] OR diabetic''[tw] OR diabetic's[tw] OR diabetica[tw] OR diabetica'[tw] OR diabetical[tw] OR diabetically[tw] OR diabeticand[tw] OR diabeticas[tw] OR diabeticassociated[tw] OR diabeticcomplications[tw] OR diabeticdyslipidemia[tw] OR diabetice[tw] OR diabeticfoot[tw] OR diabeticfoveal[tw] OR diabetichip[tw] OR diabetichip's[tw] OR diabetici[tw] OR diabeticians[tw] OR diabeticin[tw] OR diabeticke[tw] OR diabeticketoacidosis[tw] OR diabeticlike[tw] OR diabeticmen[tw] OR diabeticmothers[tw] OR diabeticnephropathy[tw] OR diabeticneuropathy[tw] OR diabetico[tw] OR diabeticorum[tw] OR diabeticos[tw] OR diabeticpatients[tw] OR diabeticpolyneuropathy[tw] OR diabeticrats[tw] OR diabeticretinopathy[tw] OR diabetics[tw] OR diabetics'[tw] OR diabetics's[tw] OR diabetics1[tw] OR diabeticses[tw] OR diabeticsts[tw] OR diabetictype[tw] OR diabeticum[tw] OR

diabeticus[tw] OR diabetid[tw] OR diabetiee[tw] OR diabeties[tw] OR diabetietes[tw] OR diabetiform[tw] OR diabetigenesis[tw] OR diabetiker[tw] OR diabetikerbund[tw] OR diabetikerkost[tw] OR diabetikern[tw] OR diabetimss[tw] OR diabetin[tw] OR diabetinol[tw] OR diabetio[tw] OR diabetiogenic[tw] OR diabetiologists[tw] OR diabetique[tw] OR diabetique'[tw] OR diabetiques[tw] OR diabetis[tw] OR diabetische[tw] OR diabetisches[tw] OR diabetised[tw] OR diabetisity[tw] OR diabetisk[tw] OR diabetiva[tw] OR diabetization[tw] OR diabetized[tw] OR diabetlic[tw] OR diabetlmss[tw] OR diabetmin[tw] OR diabeto[tw] OR diabetobiguanides[tw] OR diabetogen[tw] OR diabetogene[tw] OR diabetogenecity[tw] OR diabetogeneesis[tw] OR diabetogeneic[tw] OR diabetogeneicity[tw] OR diabetogenensis[tw] OR diabetogenes[tw] OR diabetogenes'[tw] OR diabetogenesis[tw] OR diabetogenetic[tw] OR diabetogenic[tw] OR diabetogenic'[tw] OR diabetogenicity[tw] OR diabetogenics[tw] OR diabetogenis[tw] OR diabetogenous[tw] OR diabetogens[tw] OR diabetoid[tw] OR diabetol[tw] OR diabetolgist[tw] OR diabetolgists[tw] OR diabetologem[tw] OR diabetologi[tw] OR diabetologia[tw] OR diabetologia12[tw] OR diabetologic[tw] OR diabetologica[tw] OR diabetologica'[tw] OR diabetological[tw] OR diabetologically[tw] OR diabetologico[tw] OR diabetologie[tw] OR diabetologiques[tw] OR diabetologisch[tw] OR diabetologist[tw] OR diabetologist's[tw] OR diabetologists[tw] OR diabetologists'[tw] OR diabetologits[tw] OR diabetologue[tw] OR diabetology[tw] OR diabetology'[tw] OR diabetologyteam[tw] OR diabetolytic[tw] OR diabetomobil[tw] OR diabetomobile[tw] OR diabeton[tw] OR diabetoneuropathic[tw] OR diabetoporosis[tw] OR diabetor[tw] OR diabetoral[tw] OR diabetorenal[tw] OR diabetorum[tw] OR diabetosan[tw] OR diabetotherapy[tw] OR diabetric[tw] OR diabetricorum[tw] OR diabets[tw] OR diabetss[tw] OR diabetter[tw] OR diabettics[tw] OR diabetu[tw] OR diabetus[tw]) OR NIDDM[tw] OR IDDM[tw] OR T2DM[tw] OR "Insulin Resistance"[Mesh] OR (insulin resistance[tw] OR insulin resistance,[tw] OR insulin resistances[tw] OR insulin resistancy[tw] OR insulin resistant[tw] OR insulin resistantwomen[tw]) OR (insulin sensitivities[tw] OR insulin sensitivity[tw]) OR (insulin dependence[tw] OR insulin dependency[tw] OR insulin dependend[tw] OR insulin dependent[tw] OR insulin dependenta[tw] OR insulin dependently[tw] OR insulin dependents[tw])) AND ("Influenza, Human"[Mesh] OR (influenza[tw] OR influenza'[tw] OR influenza's[tw] OR influenza3[tw] OR influenzaa[tw] OR influenzae[tw] OR influenzae's[tw] OR influenzaedisease[tw] OR influenzaeinfections[tw] OR influenzaelike[tw] OR influenzaemurium[tw] OR influenzaepidemic[tw] OR influenzaeprotein[tw] OR influenzaeresistance[tw] OR influenzaetype[tw] OR influenzai[tw] OR influenzain[tw] OR influenzainfection[tw] OR influenzal[tw] OR influenzalike[tw] OR influenzanet[tw] OR influenzano[tw] OR influenzaoltas[tw] OR influenzaran[tw] OR influenzas[tw] OR influenzasmall[tw] OR influenzata[tw] OR influenzatiring[tw] OR influenzatop[tw] OR influenzavirus[tw] OR influenzaviruses[tw]) OR flu[tw] OR ILI[tw]) AND ("Influenza Vaccines"[Mesh] OR "Influenza Vaccines"[nm] OR (vaccin[tw] OR vaccin'[tw] OR vaccina[tw] OR vaccinable[tw] OR vaccinacion[tw] OR vaccinaction[tw] OR vaccinae[tw] OR vaccinaemia[tw] OR vaccinaio[tw] OR vaccinaion[tw] OR vaccinair[tw] OR vaccinal[tw] OR vaccinale[tw] OR vaccinales[tw] OR vaccinali[tw] OR vaccinalis[tw] OR vaccinam[tw] OR vaccinant[tw] OR vaccinarian[tw] OR vaccinarsi[tw] OR vaccinary[tw] OR vaccinat[tw] OR vaccinatable[tw] OR vaccinate[tw] OR vaccinate'[tw] OR vaccinate'litopenaeus[tw] OR vaccinate's[tw] OR vaccinated[tw] OR vaccinated'[tw] OR vaccinated'against[tw] OR vaccinates[tw] OR vaccinates'[tw] OR vaccinatfon[tw] OR vaccinatin[tw] OR vaccinating[tw] OR vaccinating'[tw] OR vaccinatio[tw] OR vaccinatiob[tw] OR vaccination[tw] OR vaccination'[tw] OR vaccination's[tw] OR vaccination60[tw] OR vaccinationa[tw] OR vaccinational[tw] OR vaccinationdagger[tw] OR vaccinationem[tw] OR vaccinationis[tw] OR

vaccinationism[tw] OR vaccinationists[tw] OR vaccinationists'[tw] OR vaccinations[tw] OR vaccinations'[tw] OR vaccinationscar[tw] OR vaccinationwas[tw] OR vaccinatkon[tw] OR vaccinaton[tw] OR vaccinator[tw] OR vaccinator'[tw] OR vaccinator's[tw] OR vaccinators[tw] OR vaccinators'[tw] OR vaccinatum[tw] OR vaccinaux[tw] OR vaccinazione[tw] OR vaccinazioni[tw] OR vaccincated[tw] OR vaccincation[tw] OR vaccince[tw] OR vaccinces[tw] OR vaccincine[tw] OR vaccindtion[tw] OR vaccine[tw] OR vaccine'[tw] OR vaccine's[tw] OR vaccine1[tw] OR vaccine13[tw] OR vaccine2014[tw] OR vaccinea[tw] OR vaccinead5[tw] OR vaccineand[tw] OR vaccineas[tw] OR vaccineassociated[tw] OR vaccineatd[tw] OR vaccinecad[tw] OR vaccinechallenged[tw] OR vaccined[tw] OR vaccineda[tw] OR vaccinedelivery[tw] OR vaccinediluent[tw] OR vaccinee[tw] OR vaccinee'[tw] OR vaccinee's[tw] OR vaccinees[tw] OR vaccinees'[tw] OR vaccinefor[tw] OR vaccineforme[tw] OR vaccineformis[tw] OR vaccinefrom[tw] OR vaccinein[tw] OR vaccineinduced[tw] OR vaccinelike[tw] OR vaccinelymph[tw] OR vaccinemediated[tw] OR vaccinemia[tw] OR vaccineontology[tw] OR vaccineotherapy[tw] OR vaccinepressure[tw] OR vaccinepreventable[tw] OR vacciner[tw] OR vaccinerate[tw] OR vaccines[tw] OR vaccines'[tw] OR vaccines''[tw] OR vaccines'major[tw] OR vaccines4kids[tw] OR vaccinesafety[tw] OR vaccinesagainst[tw] OR vaccinesan[tw] OR vaccinescompared[tw] OR vaccineselection[tw] OR vaccineshoppe[tw] OR vacciness[tw] OR vaccinessummary[tw] OR vaccinesthe[tw] OR vaccineswork[tw] OR vaccinetherapy[tw] OR vaccineto[tw] OR vaccinety21a[tw] OR vaccineurin[tw] OR vaccineusing[tw] OR vaccinex[tw] OR vaccinforme[tw] OR vaccini[tw] OR vaccinia[tw] OR vaccinia'[tw] OR vaccinia's[tw] OR vacciniae[tw] OR vacciniaimmune[tw] OR vaccinial[tw] OR vaccinialike[tw] OR vaccinias[tw] OR vacciniate[tw] OR vacciniation[tw] OR vacciniavirus[tw] OR vaccinic[tw] OR vaccinica[tw] OR vaccinicide[tw] OR vaccinie[tw] OR vaccinieae[tw] OR vaccinietea[tw] OR vaccinifiers[tw] OR vaccinifolia[tw] OR vaccinifolium[tw] OR vacciniform[tw] OR vacciniforme[tw] OR vacciniformia[tw] OR vacciniformis[tw] OR vaccinii[tw] OR vacciniicola[tw] OR vacciniicorymbosi[tw] OR vacciniifolia[tw] OR vacciniifolium[tw] OR vaccininin[tw] OR vaccininum[tw] OR vaccinio[tw] OR vaccinioideae[tw] OR vaccinioides[tw] OR vacciniola[tw] OR vacciniosa[tw] OR vaccinists[tw] OR vacciniuim[tw] OR vaccinium[tw] OR vacciniumashei[tw] OR vacciniumcorymbosum[tw] OR vacciniums[tw] OR vacciniumvitis[tw] OR vacciniumxintermedium[tw] OR vacciniun[tw] OR vaccinization[tw] OR vaccinization'[tw] OR vaccinized[tw] OR vaccinnated[tw] OR vaccinnes[tw] OR vaccinnet[tw] OR vaccinnet'[tw] OR vaccinnet's[tw] OR vaccinniforme[tw] OR vaccino[tw] OR vaccinocidin[tw] OR vaccinoderma[tw] OR vaccinogen[tw] OR vaccinogenic[tw] OR vaccinogenicity[tw] OR vaccinogeno[tw] OR vaccinogens[tw] OR vaccinoid[tw] OR vaccinoids[tw] OR vaccinol[tw] OR vaccinologic[tw] OR vaccinological[tw] OR vaccinologie[tw] OR vaccinologist[tw] OR vaccinologist's[tw] OR vaccinologists[tw] OR vaccinologists'[tw] OR vaccinology[tw] OR vaccinology'[tw] OR vaccinolosa[tw] OR vaccinols[tw] OR vaccinome[tw] OR vaccinomic[tw] OR vaccinomics[tw] OR vaccinomics'[tw] OR vaccinoprevention[tw] OR vaccinoprophylactic[tw] OR vaccinoprophylaxis[tw] OR vaccinoscopie[tw] OR vaccinoside[tw] OR vaccinosis[tw] OR vaccinosis'[tw] OR vaccinostercus[tw] OR vaccinostyle[tw] OR vaccinostyles[tw] OR vaccinotherapy[tw] OR vaccinothrapy[tw] OR vaccinovigilance[tw] OR vaccinovigilanza[tw] OR vaccinovirus[tw] OR vaccinoyl[tw] OR vaccins[tw] OR vaccint[tw] OR vaccintation[tw] OR vaccinted[tw] OR vaccintes[tw] OR vaccintion[tw] OR vaccinuim[tw] OR vaccinum[tw] OR vaccinyl[tw]) OR (immuniz[tw] OR immunizability[tw] OR immunizable[tw] OR immunizaion[tw] OR immunizaition[tw] OR immunizaiton[tw] OR immunizarion[tw] OR immunizate[tw] OR immunizated[tw] OR immunizati[tw] OR immunizating[tw] OR

immunizatino[tw] OR immunizatiom[tw] OR immunization[tw] OR immunization'[tw] OR immunization''[tw] OR immunization's[tw] OR immunizational[tw] OR immunizationation[tw] OR immunizationed[tw] OR immunizationmayinduce[tw] OR immunizationor[tw] OR immunizations[tw] OR immunizations'[tw] OR immunizationshowed[tw] OR immunizaton[tw] OR immunizator[tw] OR immunizatory[tw] OR immunizd[tw] OR immunize[tw] OR immunize'[tw] OR immunizeca[tw] OR immunized[tw] OR immunized'[tw] OR immunizedshowed[tw] OR immunizer[tw] OR immunizers[tw] OR immunizers'[tw] OR immunizes[tw] OR immunizied[tw] OR immunizig[tw] OR immunizing[tw] OR immunizing'[tw] OR immunizinged[tw] OR immunizingly[tw] OR immunizition[tw] OR immunizor[tw] OR immuniztion[tw] OR immuniztions[tw]) OR (immunis[tw] OR immunisability[tw] OR immunisable[tw] OR immunisare[tw] OR immunisated[tw] OR immunisatio[tw] OR immunisation[tw] OR immunisation'[tw] OR immunisation's[tw] OR immunisationprotocol[tw] OR immunisations[tw] OR immunisaton[tw] OR immunisazions[tw] OR immuniscintigraphic[tw] OR immunise[tw] OR immunised[tw] OR immunised'[tw] OR immuniser[tw] OR immunisers[tw] OR immunisers'[tw] OR immunises[tw] OR immunished[tw] OR immunisierungstraining[tw] OR immunising[tw] OR immunising'[tw] OR immunisolated[tw] OR immunistochemical[tw] OR immunistochemistry[tw] OR immunistumulatory[tw] OR immunisuppression[tw] OR immuniszed[tw]) OR (immunogen[tw] OR immunogen'[tw] OR immunogen's[tw] OR immunogenable[tw] OR immunogenci[tw] OR immunogencity[tw] OR immunogene[tw] OR immunogenecities[tw] OR immunogenecity[tw] OR immunogeneic[tw] OR immunogeneicity[tw] OR immunogeneity[tw] OR immunogenes[tw] OR immunogeneses[tw] OR immunogenesis[tw] OR immunogenesity[tw] OR immunogenet[tw] OR immunogenetherapy[tw] OR immunogenetic[tw] OR immunogenetica[tw] OR immunogenetical[tw] OR immunogenetically[tw] OR immunogeneticist[tw] OR immunogeneticist's[tw] OR immunogeneticists[tw] OR immunogenetics[tw] OR immunogenetics'[tw] OR immunogenetique[tw] OR immunogenetists[tw] OR immunogenety[tw] OR immunogeni[tw] OR immunogenic[tw] OR immunogenic'[tw] OR immunogenical[tw] OR immunogenically[tw] OR immunogenice[tw] OR immunogenicite[tw] OR immunogenicities[tw] OR immunogenicitiy[tw] OR immunogenicity[tw] OR immunogenicity'[tw] OR immunogeniciy[tw] OR immunogenicproperties[tw] OR immunogenics[tw] OR immunogenictiy[tw] OR immunogenictumor[tw] OR immunogenicty[tw] OR immunogenicum[tw] OR immunogeniecity[tw] OR immunogenisity[tw] OR immunogenities[tw] OR immunogenity[tw] OR immunogenization[tw] OR immunogenize[tw] OR immunogenized[tw] OR immunogenocity[tw] OR immunogenodiagnosis[tw] OR immunogenodiagnostic[tw] OR immunogenome[tw] OR immunogenomic[tw] OR immunogenomics[tw] OR immunogenomics'[tw] OR immunogenoside[tw] OR immunogenotype[tw] OR immunogenotyped[tw] OR immunogenotypes[tw] OR immunogenotypic[tw] OR immunogenotypical[tw] OR immunogenotypically[tw] OR immunogenotyping[tw] OR immunogenous[tw] OR immunogens[tw] OR immunogens'[tw] OR immunogentic[tw] OR immunogenticity[tw] OR immunogenuity[tw] OR immunogenum[tw]) OR (immunit[tw] OR immunitaire[tw] OR immunitaires[tw] OR immunital[tw] OR immunitaly[tw] OR immunitarian[tw] OR immunitary[tw] OR immunitas[tw] OR immunitat[tw] OR immunite[tw] OR immunited[tw] OR immunites[tw] OR immunities[tw] OR immunitin[tw] OR immunition[tw] OR immunitiy[tw] OR immunitor[tw] OR immunitortrade[tw] OR immunitory[tw] OR immunitry[tw] OR immuniturbidimetric[tw] OR immunity[tw] OR immunity'[tw] OR immunity''[tw] OR immunity's[tw] OR immunity1[tw] OR immunity2[tw] OR immunity40[tw] OR immunity41[tw] OR immunityagainst[tw] OR immunityand[tw] OR immunityassociated[tw] OR immunitycan[tw] OR

immunitymytilus[tw] OR immunitywhilst[tw])) AND "humans"[MeSH Terms]
**Cochrane Library**<br>#1 MeSH descriptor: [Metabolic Diseases] explode all trees<br>#2 MeSH descriptor: [Diabetes Mellitus] explode all trees<br>#3 MeSH descriptor: [D007333] explode all trees<br>#4 #1 OR #2 OR #3<br>#5 diabet* OR (insulin AND (resistan* OR sensitiv* OR dependen*)) OR niddm OR iddm OR t2dm OR "metabolic syndrome x" OR "syndrome x"78190<br>#6 #4 OR #5<br>#7 MeSH descriptor: [Influenza Vaccines] explode all trees<br>#8 Influenza Vaccines OR vaccin* OR immun*104947<br>#9 #7 OR #8<br>#10 #6 AND #9<br>#11influenza<br>#12 #10 AND #11
**Embase**<br>The following search strings were used to search for relevant articles in Embase.<br>('metabolic disorder'/exp OR 'metabolic disorder' OR 'metabolic disease' OR 'metabolic diseases' OR 'metabolic disorders' OR 'diabetes mellitus'/exp OR diabet* OR niddm OR iddm OR t2dm OR 'insulin resistance'/exp OR 'insulin resistance' OR (insulin AND (resistan* OR sensitiv* OR dependen*)) OR 'metabolic syndrome x'/exp OR 'metabolic syndrome x' OR 'syndrome x') AND ('influenza'/exp OR 'influenza' OR 'influenza virus'/exp OR 'influenza virus') AND ('influenza vaccine'/exp OR 'influenza vaccine' OR vaccin* OR immun*) AND [humans]/lim
